# Technical Problems and Ethical Concerns Regarding Gene Editing in Human Germlines and Embryos

**DOI:** 10.18502/jri.v24i3.13270

**Published:** 2023

**Authors:** Mohammad Reza Sadeghi

In vitro fertilization was initially introduced by Patrick Steptoe and Robert Edwards in 1978 in England with the aim of treating infertility of Lesley Brown due to damage or blockage of her fallopian tubes. Later, a wide range of techniques were developed for treatment of infertility such as IVF, GIFT, ZIFT, SUZI, ICSI, TESE, and PESA based on its many potential causes. According to the global ART monitoring report, presented by David Adamson on behalf of International Committee for Monitoring Assisted Reproductive Technologies (ICMART) at the annual meeting of ESHRE in 2023, more than 12 million babies have been born around the world since the first IVF birth about 45 years ago. One such example of the ever-escalating trend in application and expansion of ART is the 3.5 million IVF cycles in 2019, that led to the birth of 750,000 babies. It is estimated that by the year 2100, the number of babies born from IVF will reach 400 million or 3% of the world’s population (1).

In addition, in vitro fertilization of human and other mammalian oocytes and embryo development to blastocyst stage result in IVF advancement and introduction of treatment possibilities in other fields of medicine and biology such as embryonic stem cells, cloning, genetic manipulations of animal embryos (knockout and knock-in animals), and preimplantation genetic testing of human embryo for monogenic disorders (PGT-M) or aneuploidy (PGT-A) or structural rearrangements (PGT-SR). Therefore, the use of PGT techniques added to the applications of IVF for fertile couples who can produce numerous embryos despite carrying defective genes or suffering from genetic diseases; in fact, PGT, evolving as an essential procedure in ART, paves the way for successful IVF procedures by selecting unaffected embryos and transferring them to the uterus. Also, these methods are frequently used to select euploid embryos for fertile women with recurrent pregnancy loss (RPL) and infertile women with repeated implantation failures (RIF). Moreover, advances in gene technology and application of cutting-edge approaches such as NGS, CGH array, and SNP array improved accuracy and efficiency of PGT. However, the main problem of PGT is that all affected embryos should be discarded with no ability for editing their genetic errors; this problem is more evident when all tested embryos are affected and there is no possibility to take any embryos in other cycles (2).

The first genome editing technology was introduced in 1984 with successful insertion of the new genetic material into pluripotent hematopoietic stem cells in mice, which showed that the virus could transfer desired genes into the target cells. This was the starting point for the possibility of gene editing and recent gene therapy technology for a wide range of genetic and non-genetic diseases. The first effective gene therapy was performed in a 4-year-old girl with severe combined immunodeficiency (SCID) due to adenosine deaminase (ADA) deficiency in 1990. The Necker–Enfants Malades Hospital reported the first clinical trial of gene therapy for SCID-X1 in France in 2000, but five of 20 treated children died of cancer. The viral vector for gene insertion into T cells activated proto-oncogene and led to leukemia. At the same time, an 18-year-old boy died in the United States following gene therapy for a rare metabolic disorder of ornithine transcarbamylase (OTC) deficiency. In this tragic story that shocked the world, the viral vector induced a lethal immune response with multiple organ failure and brain death. Therefore, the flames of fear, hope, and desire for opening the horizons in treatment of many rare genetic diseases has been extinguished and the incident halted all gene therapy trials (3). Therefore, research switched its focus from old methods of using viral vectors to finding new technologies. This field has fundamentally changed in less than ten years following abandonment of gene editing research. Emergence of several new tools including ZFNs, TALENs and CRISPR/Cas9 has translated the idea of gene editing into clinical practice. CRISPR/Cas9, the so-called “genetic scissors”, has many advantages over ZFNs and TALENs due to its great accuracy, excellent efficiency, and high specificity. Consequently, only eight years later, Emmanuelle Charpentier and Jennifer A. Doudna were jointly awarded the Nobel Prize in Chemistry (2020) for the development of CRISPR/Cas9 which allows the researchers to precisely cut and edit human genome (4). After confirming the effectiveness of CRISPR/Cas9 through experimental studies, the first *ex vivo* clinical trial using CRISPR/Cas9 to inactivate the PD-1 gene in blood cells was conducted in China in 2016. The engineered cells returned to circulation to attack non-small cell lung cancers (NSCLCs). CRISPR/Cas9 was subsequently used to treat a patient with sickle cell anemia in 2019 in the United State. Despite solid evidence on the efficiency of this system, following publication of the preliminary results of clinical trials, there were widespread discussions and concerns about the safety, and ethical and technical challenges in CRISPR/Cas9 manipulations of human genome. Numerous conferences and articles published in this field basically recommended prohibition of using genetic editing in human embryonic cells. In fact, the research on CRISPR/Cas9 technology is still in its infancy and its lack of efficiency and specificity raises uncertainties regarding the application of the technology in human embryo gene editing (3, 5).

Despite the concern of the scientific community and societies on prohibition of gene editing in human embryos, a Chinese scientist, He Jiankui, used CRISPR/Cas9 system through in vitro fertilization (IVF) process to delete the gene copies responsible for the HIV receptor in human immune cells. He publicized the birth of two girls in 2018 while global protests and public pressure caused him to be imprisoned for 3 years. A few months later, a second scientific scandal was revealed. Denis Rebrikov, a Russian scientist who initially planned to edit the same CCR5 gene responsible for HIV receptor on human cells, changed the target gene to GJB2 as the single most frequent cause of genetic hearing loss due to the pressure of scientific community and protests (5). At present, the legal frameworks for gene editing of human germline and embryo in different countries are not the same. Therefore, the gene editing of the embryo for non-reproduction purposes is allowed in at least 11 countries, including China, United States, and UK. Nineteen countries, including Belarus, Canada, Sweden, and Switzerland have banned gene editing trials on human embryo. In other countries (including Russia), a neutral position is adopted (5).

Recently, during congress of the European Society of Human Reproduction and Embryology (ESHRE) in 2023 in Copenhagen, Nada Kubikova presented the findings of her research team at Oxford University. The primary objective of their research was to determine the feasibility of using CRISPR/Cas9 to correct genetic errors in human preimplantation embryos, as well as to investigate the potential short-term and long-term effects of utilizing this tool for genome editing of early embryos. In their study, 84 embryos were produced using donated sperm and oocytes through ICSI. Following embryo development for 60 *hr*, blastomeres were subjected to whole genome amplification (WGA) and next-generation sequencing (NGS) to identify segmental aneuploidy and evaluate DNA repair after DNA breakage through CRISPR/Cas9 system. In total, 53 double-strand breaks were created by CRISPR/Cas9, of which 32 (60%) were repaired and 21 (40%) remained unrepaired leading to segmental aneuploidy in the embryo. Therefore, they concluded that CRISPR/Cas9 has the ability to induce DNA breakage with high efficiency but most of embryos are unable to repair DNA damage (6).

Therefore, despite the potential of CRISPR/Cas9 to revolutionize human gene editing, there are many drawbacks and ethical concerns surrounding its application in human embryos and germlines. Its recent potential shortcomings including off-target effects, increased mosaicism, the unknown nature of all genes and genetic diseases, and further ethical concerns call for caution in using CRISPR/Cas9 technology in embryos and germline gene editing. Therefore, it is necessary to conduct further research on the risks and potential benefits of gene editing in human embryos and germlines before proceeding with clinical applications.

## References

[1] European society of human reproduction and embryology [Internet].Belgium: European society of human reproduction and embryology: Focus on reproduction At least 12 million babies’ since the first IVF birth in 1978; 2023 Jun 28 [Ccited 2023 Jul 29]; [about 2 screens]. Availble from: https://www.focusonreproduction.eu/article/ESHRE-News-COP23_adamson

[2] Greco E Litwicka K Minasi MG Cursio E Greco PF Barillari P. Preimplantation genetic testing: where we are today. Inte J Mol Sci. 2020;21(12):4381. 10.3390/ijms21124381 PMC7352684 32575575

[3] Secord E Hartog NL. Review of treatment for adenosine deaminase deficiency (ADA) severe combined immuno-deficiency (SCID). Ther Clin Risk Manag. 2022;18:939–44. 36172599 10.2147/TCRM.S350762 PMC9512634

[4] Farhud DD Zarif-Yeganeh M. CRISPR pioneers win 2020 nobel prize for chemistry. Iran J Public Health. 2020;49 (12):2235–9. 34178729 10.18502/ijph.v49i12.4800 PMC8215069

[5] Gostimskaya I. CRISPR–Cas9: A History of Its Discovery and Ethical Considerations of Its Use in Genome Editing. Biochemistry (Moscow). 2022;87(8):777–88. 36171658 10.1134/S0006297922080090 PMC9377665

[6] Kubikova N Esbert M Titus S Coudereau C Savash M Fagan J O-075 Deficiency of DNA double-strand break repair in human preimplantation embryos revealed by CRISPR-Cas9. Hum Reprod. 2023;38(Supplement_1):dead093-089.

